# Surface defect detection competition with a bio-inspired vision sensor

**DOI:** 10.1093/nsr/nwad130

**Published:** 2023-05-09

**Authors:** Anjing Guo, Jupo Ma, Renwei Dian, Fuyan Ma, Jinjian Wu, Shutao Li

**Affiliations:** School of Robotics, Hunan University, China; School of Artificial Intelligence, Xidian University, China; Pazhou Laboratory (Huangpu), China; School of Robotics, Hunan University, China; College of Electrical and Information Engineering, Hunan University, China; Key Laboratory of Visual Perception and Artificial Intelligence of Hunan Province, China; School of Artificial Intelligence, Xidian University, China; Pazhou Laboratory (Huangpu), China; School of Robotics, Hunan University, China; College of Electrical and Information Engineering, Hunan University, China

## Abstract

This paper reports the background and results of the Surface Defect Detection Competition with Bio-inspired Vision Sensor, as well as summarizes the champion solutions, current challenges and future directions.

## INTRODUCTION

Surface defects are inevitable during the product manufacturing process, such as impurities, scratches and dirt. Defects not only reduce the aesthetics and comfort of products, but may also degrade the functional performance. Manufacturers are very concerned about inadequate defect detection because of potential economic and reputation losses. Thus, it is significant to study automatic defect detection methods.

Existing machine vision–based defect detection mostly adopts a traditional CCD or CMOS vision sensor (VS) for imaging. However, in real-world production lines, due to dynamic environments, traditional VS encounters difficulties in meeting the demands of defect detection, especially in modern high-precision product manufacturing.

The novel bio-inspired vision sensor [1] works on the principle of energy difference, which is also known as the dynamic vision sensor or event camera. Different from the traditional VS that outputs full frames at a fixed frame rate, the bio-inspired VS measures per-pixel brightness changes asynchronously and outputs a stream of events. Each event encodes the coordinate, time and brightness attributes. The bio-inspired VS has the characteristics of high temporal resolution, low latency and high dynamic range. It has unique advantages in weak defect imaging. For example, in the challenging scenarios of small and weak defects, the bio-inspired VS still captures the defective areas effectively.

### Challenge

Bio-inspired VS-based defect detection has the following challenges.

- *New data format*. The outputs of bio-inspired VS are not frames, but a series of sparse and asynchronous events. Existing mature frame-based algorithms cannot be directly applied.
- *A lot of noise*. Because of the instability of pixel circuits and light sources, the event steam contains a lot of noisy events.
- *Small and weak defect*. In modern high-precision products, the defective areas are usually small and weak, for example, spot defects with a diameter less than 200 μm. Small defects and serious noise result in low signal-to-noise ratio.

In order to promote the development of defect detection, this competition focuses on the surface defect detection on aluminum substrates captured by bio-inspired VS. An aluminum substrate is a high-precision industrial product, which is an important component in the production of hard disks. Surface defects seriously degrade the performance of aluminum substrates. The teams were required to determine the bounding boxes and defect categories of defective areas.

### Competition details

#### Dataset

A total of 200 event streams were recorded in this competition, containing both defective and non-defective aluminum substrates. The dataset includes three common defect types: spot defect, scratch defect and stain defect. The minimum diameter of spot defects was less than 200 μm and the minimum depth of scratch defects was less than 10 μm. During the recording, the bio-inspired VS was fixed and aluminum substrates moved at a constant speed. All samples were divided into three subsets: 120 for TrainSet, 30 for TestA and 50 for TestB. TestB served as the final test data.

#### Evaluation metric

The performance evaluation metric is set as


(1) \begin{eqnarray*} \mathrm{Score} = 0.2\times \mathrm{ACC} + 0.8\times \mathrm{mAP}, \end{eqnarray*}

with ACC the classification accuracy of aluminum substrates and mAP the mean average precision [2]. All samples were divided into five categories: spot defect, scratch defect, stain defect, no defect and multiple defects. If one aluminum substrate contained more than one defect, it belonged to the ‘multiple defect’ category. In this competition, the IOU thresholds were set as 0.4, 0.5 and 0.6 to compute the mAP.

## THE SOLUTION OF THE CHAMPION TEAM

**Figure 1.**
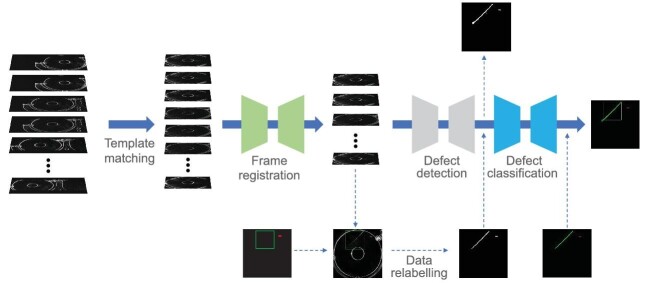
The framework of the champion team’s solution. The proposed method consists of five main steps: template matching, frame registration, data relabeling, defect detection and defect classification.

This section introduces the champion team’s solution, which transforms the task into a semantic segmentation problem and proposes a two-stage segmentation model for defect detection with limited training data. Specifically, an event stream is first converted to a set of frames with a fixed event window. Then an industrial product is extracted from each frame with a set of convolutional templates. In order for these pieces of industrial products to be considered as a whole, a U-shaped [3] frame registration network is further constructed to produce pixel-wise offsets for each industrial product frame, which can be utilized for aligning the frame with the convolutional templates. Next, the defect detection problem is transformed into an image segmentation problem and the training data are relabeled. The advantage of this transformation is that image segmentation is a dense prediction task, which is more suitable for limited training data. Furthermore, two lightweight U-shaped networks are introduced for the image segmentation task, where the first sub-network is for preliminary defect location, and the second sub-network is for accurate defect classification. The whole flow of the method is shown in Fig. 1.

### Template matching

Template matching is the first step of this solution, which extracts the industrial product pieces from the converted frames. As shown in Fig. S1 within the online supplementary material, the fixed number of events for converting an event stream to frames is set to 50 000. A set of convolutional templates is constructed for the industrial product, comprising two concentric circles, one large and one small. These two templates are like two kernels of the convolutional layer, and the matching process is similar to the convolution process. The obtained features are the probability maps of the industrial product’s center. After selecting appropriate thresholds, the center of the industrial product can be obtained and the industrial product can be extracted from these frames.

### Frame registration

During the imaging process, the industrial product is moving in translation, which means that its position relative to the event camera is constantly changing, resulting in a slight distortion of the industrial product in different frames. Hence, in order for the extracted pieces of industrial product to be considered as a whole, it is necessary to introduce a frame registration network to align them.

As shown in Fig. S2 within the online supplementary material, the frame registration network is a U-shaped network. The encoder of the network consists of four convolutional blocks, where the first convolutional layer has 32 5 × 5 kernels with dilation rate 2 and the second convolutional layer has 32 5 × 5 kernels with stride 2, with the max-pooling layer only included in the first two blocks. The extracted product piece and the combined template are concatenated and sent to the encoder. The output of the encoder is the downsampled offset maps of the extracted product piece in two directions. The decoder of the network resizes the offset maps to the same size as the input product piece, which are then utilized for resampling the product piece to align it with the combined template. The resampling process is implemented by a bilinear interpolation operation [4], and it is derivable.

The loss function of the frame registration network is based on a masked mean absolute error (MAE), which can be described as


(2) \begin{eqnarray*} {L_1} &=& \mathrm{MAE}(Y \bullet \hat{Y},\hat{Y})\\ && +\, \alpha \bullet \mathrm{TV(offsets),} \end{eqnarray*}

with *Y* the resampled frame and $\hat{Y}$ the combined template. Here TV(offsets) means that ℓ_1_ − TV is used to constrain the pixel-wise offset maps, and α is set to 0.01.

### Data relabeling

After the above-mentioned operations, the event stream can be analyzed as a whole, which eliminates the need to label images frame by frame, greatly reducing the amount of labeling. However, a total of 120 event streams makes it impossible to effectively train the conventional detection models such as YOLO [5] and faster-RCNN [6]. The core idea of this solution is transforming the detection task into a semantic segmentation framework to combat insufficient training data. All registered product pieces are averaged and the data are relabeled in a dense pixel-wise labeling way. As shown in Fig. S3 within the online supplementary material, after the frame registration, clearer structures of the industrial product can be obtained, resulting in more accurate and reasonable labels.

### Defect detection

In this section, a lightweight U-shaped network is constructed for preliminary defect detection, where all defects are treated as being of the same class. The structure of the network is shown in Fig. S4 within the online supplementary material. As can be observed, since each event stream has a different imaging duration, it is resized to 50 frames in the channel dimension. These frames and the combined template are then stacked and fed into the network. The desired output is binarized labels.

The loss function of this part is a weighted binary cross-entropy loss, which can be expressed as


(3) \begin{eqnarray*} {L_2} &=& -w \bullet y \log (\widetilde{y})\\ && -\, (1-y) \log (1-\widetilde{y}), \end{eqnarray*}

where *w* is the weight of the defect class and set to 10.0.

### Defect classification

Another sub-network is constructed for accurate defect classification, which has a similar structure to the previous detection network. As shown in Fig. S5 within the online supplementary material, the input of this network is based on the previous detection network. The feature maps of the penultimate layer of the detection network are extracted. Then the extracted feature maps are concatenated with the predicted results and sent to this classification network. This network’s job is like coloring an image by assigning different colors (different defect classes) to the predicted defects.

The inspiration for designing this detection solution comes from the famous faster-RCNN; hence, the procedure for training the detection sub-network and classification sub-network is similar to training faster-RCNN. Specifically, the defect detection network is trained first, then its parameters are frozen and the classification network is trained. The loss function of this part is the weighted cross-entropy, that is,


(4) \begin{eqnarray*} {L_3} = -\frac{1}{n} \sum _{i=1}^n \sum _{j=1}^k w_{j} \bullet y_{i j} \log (\widetilde{y}_{i j}), \end{eqnarray*}

where *w_j_* is the weight of class *j*. It is set to 60.0 for the spot class, 10.0 for the scratch class and 25.0 for the stain class.

### Post-processing

Similar to the non-maximum suppression step of faster-RCNN, there was also a need to post-process the predicted results. Specifically, the discontinuity of segmentation results is one of the challenges in semantic segmentation, such as discontinuous roads or blood vessels. These discontinuous results often lead to over-detection or missing detection in generating the bounding boxes. To tackle this, multi-scale probabilities are utilized in post-processing the output results of the above-mentioned networks, as displayed in Fig. S6 within the online supplementary material.

### Results

This section reports the results of the champion team’s solution. Table S1 within the online supplementary material lists the results of the top eight finalist teams. The champion team’s solution obtains the highest ACC, mAP and the final weighted score of (0.2ACC+0.8mAP), achieving first place in the final challenge results.

As shown in Table S2 within the online supplementary material, all three deep models of the champion team’s solution are lightweight, which ensures the real-time performance of this solution in practice.

## COMMENTS ON THE CHAMPION TEAM’S SOLUTION

The champion’s solution proposes two novel and interesting techniques to solve the weak defect detection: a task transformation method and a temporal information fusion method. It has the following advantages.

1. By transforming the detection problem into a segmentation problem that is a pixel-wise prediction task, the champion’s solution is effective in solving the limitation of small training data size.
2. The solution proposes a region-of-interest (ROI) registration-based method for temporal information fusion. This technique makes full use of the prior characteristics of the aluminum substrate, which is robust enough to deal with noisy events, object discontinuities and object deformations. Additionally, under the premise of the segmentation task, ROI registration also alleviates the complexity of relabeling the training data.
3. The champion team’s solution achieves the best results on both ACC and mAP. ACC and mAP are respectively 2.6% and 4.1% higher than the second-place team. The final score achieves 0.36, which is 5.9% higher than the second-place team and 38.5% higher than the third-place team. In particular, the 0.78 ACC ensures that the algorithm could be used to filter out the defective aluminum substrates in practical applications.
4. The champion team designed a lightweight network module that is easy to implement in micro computation systems and also reduces application cost.

To sum up, the champion team’s solution not only achieves the highest score on the competition dataset, but is also innovative and has great application potential in weak defect detection on aluminum substrate.

## CONCLUSION AND FUTURE DIRECTION

The competition aims to promote the development of defect detection algorithms. To solve the limitation of traditional frame-based VS, a novel bio-inspired VS was used for defect imaging. Different teams came up with a number of novel algorithms to process this new visual signal, which greatly promoted the development of bio-inspired VS-based defect detection. Despite the significant success of the competition, there are some possible directions for future improvements.

- The champion’s solution uses two weighted cross-entropy loss functions for training the model; hence, the weights of different defect classes should be better tuned. Additionally, the accumulative error of these steps should be considered, which will make the output of the model more robust.
- The amount of data in this competition is relatively small. In real applications, more defective samples could be collected to increase the robustness of detection models.
- Bio-inspired VS can be applied to defect detection of more industrial products. Thus, more general, efficient and effective event processing methods are required.
- When dealing with asynchronous events—except CNN-based methods that convert the events into image-like tensors—point cloud methods, graph neural networks or spike neural networks could also be adopted to make full use of the asynchronous properties of events.

## Supplementary Material

nwad130_Supplemental_File
