# Supplementary material for: Surface defect detection competition with a bio-inspired vision sensor
Source: Natl Sci Rev. 2023 May 9;10(6):nwad130. doi: 10.1093/nsr/nwad130 (PMC10281496; doi:10.1093/nsr/nwad130)
Supplement: nwad130_Supplemental_File [file nwad130_supplemental_file.pdf]

PERSPECTIVE

National Science Review
XX: NSR˙defDet˙supp, Year
doi: https://doi.org/10.1093/nsr/XXXX

Computer Science

Special Topic: AI Algorithms and Cases : To Energize Digital Economy

Supplementary File – Surface Defect Detection Competition with Bio-inspired Vision Sensor

The supplementary file presents the additional figures and tables, please refer to the main body for more detailed information. Besides, more defect examples and network architecture details are also provided in the supplementary file.

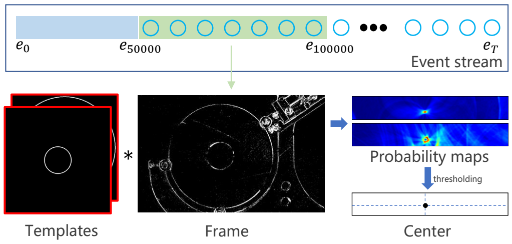

Figure 1. The template matching step.

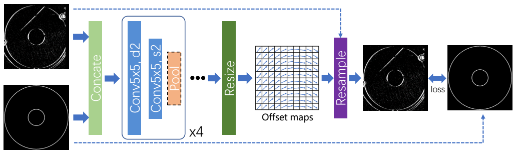

Figure 2. The structure of proposed frame registration network.

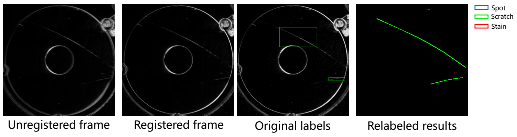

Figure 3. Visual comparisons of different labeling ways.

Network Architecture Details

Fig.2 present the structure of the proposed frame registration network, which is a U-shaped network. The encoder of the network consists of four following convolutional blocks, where the first convolutional layer has 32 5×5 kernels with dilation rate 2 and the second convolutional layer

Received: XX XX Year;
Revised: XX XX Year;
Accepted: XX XX Year

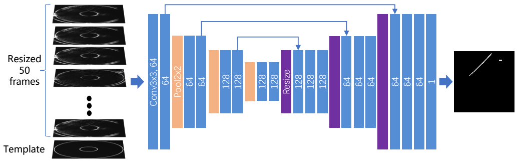

Figure 4. The structure of the proposed defect detection network.

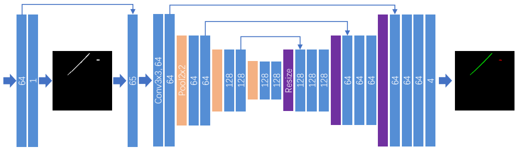

Figure 5. The structure of the proposed defect classification network.

Table 1. Final Challenge Results of first 8 teams

Team Rank	ACC	mAP	Score
1	**0.78**	**0.25**	**0.36**
2	0.76	0.24	0.34
3	0.76	0.14	0.26
4	0.54	0.16	0.24
5	0.68	0.08	0.20
6	0.46	0.13	0.20
7	0.60	0.07	0.18
8	0.60	0.00	0.12

Table 2. Model Sizes of The Three Deep Models

Model	Size
Frame Registration	0.18M
Defect Detection	1.44M
Defect Classification	1.45M

© The Author(s) Year. Published by Oxford University Press on behalf of China Science Publishing & Media Ltd. This is an Open Access article distributed under the terms of the Creative Commons Attribution License (http://creativecommons.org/licenses/by/4.0/), which permits unrestricted reuse, distribution, and reproduction in any medium, provided the original work is properly cited.

has 32 5×5 kernels with stride 2, and the max-pooling layer is only included in the first two blocks. The offset maps are generated by a convolutional layer with 2 5×5 kernels, then they are resized to the same size to input frames by the decoder part which has only an up-sampling layer.

Fig.4 presents the structure of the proposed lightweight defect detection network, which is also a U-shaped network. The encoder of the defect detection network has 4 convolutional blocks, and the number of convolution kernels in each convolution layer of these modules is 64, 64, 128, and 128 respectively, and the size of all convolution kernels is 3×3. The main target of this network is to locate the defects of products roughly.

Fig.5 presents the structure of the proposed defect classification network, which has the same structure as the proposed defect detection network. This network is connected to the defect detection network. The feature and probability maps of the detection network are concatenated as inputs, and the output of this network will classify the defect category.

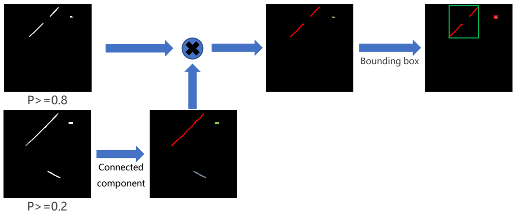

Figure 6. The flowchart of the proposed post-processing step.

Visualization of Representative Surface Defects

Fig.7 visualizes the imaging effect of bio-inspired VS on defective aluminum substrate. In the challenging scenarios of small and weak defect, the bio-inspired VS still could photograph the defective areas effectively. However, the output of bio-inspired VS is sparse, asynchronous and unstructured event streams, and the event steams contain a lot of noise. The defective areas is small and weak. Novel algorithms are required to process the events.

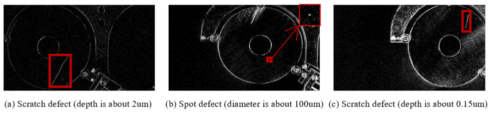

Figure 7. Defective event-images captured by the bio-inspired vision sensor.
